# *Clostridium difficile* Infection in Patients Discharged from US Short-stay Hospitals, 1996–2003^1^

**DOI:** 10.3201/eid1203.051064

**Published:** 2006-03

**Authors:** L. Clifford McDonald, Maria Owings, Daniel B. Jernigan

**Affiliations:** * Centers for Disease Control and Prevention, Atlanta, Georgia, USA

**Keywords:** *Clostridium difficile*, national rates, hospital discharges, research

## Abstract

US hospital discharges for which *Clostridium difficile*–associated disease (CDAD) was listed as any diagnosis doubled from 82,000 (95% confidence interval [CI] 71,000–94,000) or 31/100,000 population in 1996 to 178,000 (95% CI 151,000–205,000) or 61/100,000 in 2003; this increase was significant between 2000 and 2003 (slope of linear trend 9.48; 95% CI 6.16–12.80, p = 0.01). The overall rate during this period was severalfold higher in persons >65 years of age (228/100,000) than in the age group with the next highest rate, 45–64 years (40/100,000; p<0.001). CDAD appears to be increasing rapidly in the United States and is disproportionately affecting older persons. Clinicians should be aware of the increasing risk for CDAD and make efforts to control transmission of *C*. *difficile* and prevent disease.

## Abstract 2

Clinicians should be aware of the increasing risk of *C. difficile*–associated disease and make efforts to control its transmission.

*Clostridium difficile* is an anaerobic, spore-forming bacillus that produces 2 important exotoxins: toxin A, an enterotoxin, and toxin B, which is primarily a cytotoxin (*1*). *C*. *difficile* is the most commonly recognized cause of antimicrobial drug–associated diarrhea. Although *C*. *difficile*–associated disease (CDAD) is usually localized to the large bowel, where it manifests as diarrhea and pseudomembranous colitis, disease may progress to toxic megacolon, sepsis with or without intestinal perforation, and death (*2*) CDAD is increasingly recognized among residents of long-term care facilities (*3*) and even among persons living in the community (*4*); however, it most commonly affects patients in short-stay hospitals, where epidemic strains of *C*. *difficile* may be transmitted extensively both within and between facilities (*5**,**6*). Moreover, substantial excess healthcare costs and excess hospital days are associated with CDAD among short-stay hospital patients (*7**,**8*).

Several reports from individual hospitals (*9**–**11*) and a recent report primarily from the intensive care unit component of the National Nosocomial Infection Surveillance System (NNIS) (*12*) suggest that the incidence of CDAD may be increasing. However, only 90 to 340 hospitals contributed to NNIS during the study period, and these hospitals do not represent a probability sample of all US hospitals. Therefore, we analyzed national hospital discharge data to determine 1) the scope and magnitude of CDAD in US short-stay hospitals, 2) whether the rate of CDAD was indeed increasing, and if so, 3) the epidemiologic factors associated with such an increase.

## Methods

The National Hospital Discharge Survey (NHDS) is conducted annually by the National Center for Health Statistics, Centers for Disease Control and Prevention (CDC), and consists of diagnosis and demographic data collected from a national probability sample of patient discharge records (*13*). At least 90% of a panel of 500 hospitals participate, from which over 300,000 discharge records are sampled each year. Based upon the analysis weight applied to each record of this ≈1% sample, national estimates were made regarding the number and character of all nonfederal, short-stay hospital discharges. NHDS data were used to determine the number of discharges with the International Classification of Diseases, 9th Revision, Clinical Modification (ICD-9-CM) code (008.45) specific for "intestinal infection due to *Clostridium difficile*" listed as a discharge diagnosis. This code was introduced in 1993 and is the only ICD-9-CM code specific for CDAD.

National estimates of both the absolute number of discharges with CDAD listed as a discharge diagnosis, the proportions of discharges with CDAD, and the rates per 100,000 US population were determined for the years 1996–2003. Standard errors for all statistics were calculated by using SUDAAN version 7.0 (Research Triangle Institute, Research Triangle Park, NC, USA), which takes into account the complex sample design of the NHDS. A description of the software and the approach it uses have been published (*14*). Data were stratified according to whether CDAD was listed as the first-listed vs. any-listed diagnosis, patient sex, patient age, and hospital geographic region (according to US census regions), bed size, and ownership type (i.e., proprietary, state and local government, or nonprofit). The NHDS collects data on up to 7 diagnoses for each discharged patient sampled. The first-listed diagnosis is the principal diagnosis if it were specified as such on the medical record or the face sheet of the discharge summary of the patient. If the principal diagnosis is not specified, then diagnoses are listed in the order they are given. The principal diagnosis is the condition established after study as chiefly responsible for the hospitalization. Therefore, estimates for the first-listed diagnosis of CDAD refer to patients for whom CDAD is most likely the primary reason for the hospital admission. Any listed diagnosis estimates reflect discharged patients for whom CDAD was either a primary or a secondary diagnosis, including those patients who may have contracted CDAD during the current hospitalization, as well as those who acquired it by other means.

Rates of discharges with CDAD as any diagnosis over the entire study period (i.e., 1996–2003) were compared by various demographic factors using the 2-tailed Z-test based on standard errors obtained from SUDAAN. Tests of linear trend over time in rates, percentages, and numbers were performed by using a weighted least-squares regression method; p values were determined from the *t* statistic by using degrees of freedom equal to 1 less than the number of years over which trends were examined (*15*). Because the trend in overall and stratified rates changed so remarkably at the midpoint of the study period, separate trend analyses were performed for 1996–1999 and 2000–2003. Although these separate trend analysis were performed for all stratified rates presented, only the most pertinent negative findings are presented, along with all significant trend results (i.e., p<0.05).

## Results

**Figure 1:**
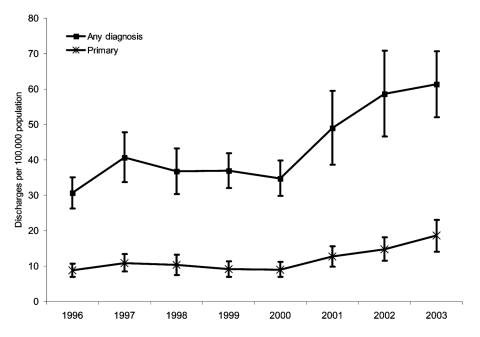
National estimates of US short-stay hospital discharges with *Clostridium difficile* listed as primary or as any diagnosis. Isobars represent 95% confidence intervals.

From 1996 through 2003, an estimated 264,000 (95% confidence interval [CI] 232,000–296,000) and 978,000 (95% CI 869,000–1,087,000) discharges for which CDAD was listed as either first or as any diagnosis, respectively. No significant trend was found in the numbers or rates of discharges with CDAD listed as either the first or any diagnosis from 1996 to 1999. In contrast, the number of discharges for which CDAD was listed as the first diagnosis increased from 25,000 (95% CI 19,000–31,000) in 2000 to 54,000 (95% CI 41,000–67,000) in 2003 (slope expressed as the average change in value per annum [*b*] = 9,000, 95% CI 5,000–13,000, p<0.001). Annual increases in the point estimates of these discharges over the precedent year were 43%, 18%, and 27% in 2001, 2002, and 2003, respectively, although the single year increases in 2002 and 2003 were not significant. Meanwhile, the number of discharges for which CDAD was listed as any diagnosis nearly doubled from 98,000 (95% CI 84,000–112,000) in 1996 to 178,000 (95% CI 151,000–205,000) in 2003 (*b* = 28,000, 95% CI 19,000–38,000, p<0.001). Annual increases in the point estimates of these discharges over the preceding year were 43%, 21%, and 5% in 2001, 2002, and 2003, respectively; again, the only significant single-year increase was in 2001. The estimated population-based rates of discharges with either a first-listed or any diagnosis of CDAD during this period are presented in Figure 1, along with the 95 CIs for these estimates. The upward trends in rates of CDAD both as first-listed (*b* = 3.1, 95% CI 1.70–4.48, p = 0.008) and as any (*b* = 9.48, 95% CI 6.16–12.80, p = 0.01) discharge diagnosis were significant between 2000 and 2003.

Based upon the estimated 250 million discharges from 1996 to 2003, the proportions of discharged patients with CDAD listed as first or any diagnosis were 0.10% (95% CI 0.092–0.11%) and 0.38% (95% CI 0.35%–0.41%), respectively. Both of these rates increased in a fashion similar to that of population-based rates shown in Figure 1. For CDAD as the first-listed discharge diagnosis, a significant upward trend was evident, from 0.08% in 2000 to 0.16% in 2003 (*b* = 0.024, 95% CI 0.014–0.0035, p = 0.02). For CDAD as any discharge diagnosis, a significant upward trend occurred from 0.27% in 2000 to 0.51% in 2003 (*b* = 0.072, 95% CI 0.048–0.095, p = 0.009). Rates of any CDAD discharge diagnosis over the entire study period (1996–2003) are shown stratified by various demographic factors in the Table; overall rates during this period were identical in male and female discharged patients.

**Table:** Overall rates of any listed CDAD discharge diagnosis by various demographic factors, 1996–2003*

Demographic factor	Point estimate of rate†	95% confidence interval†	p value
Sex			
Male	0.38%	0.34%–0.42%	
Female	0.38%	0.34%–0.42%	NS
Age group (y)			
>65	228	200–256	
45–64	40	34–45	<0.001
15–45	11	10–13	<0.001
<15	9	5–9	<0.001
Geographic region			
Northeast	68	56–79	
Midwest	49	36–61	0.03
South	36	27–45	<0.001
West	31	26–37	<0.001
Hospital size by number of beds			
<100	0.30%	0.23%–0.36%	
100–200	0.42%	0.37%–0.47%	0.004
>300	0.38%	0.35%–0.40%	0.03

*CDAD, *Clostridium difficile*–associated disease; NS, not significant. †Per 100,000 population unless otherwise indicated as the proportion (%) of hospital discharges.

**Figure 2:**
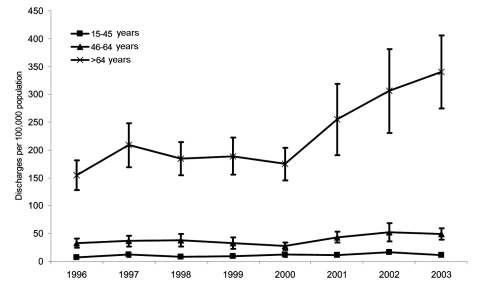
Rates of US short-stay hospital discharges with *Clostridium difficile* listed as any diagnosis, by age. Isobars represent 95% confidence intervals. Because of low rates and the resulting uncertainty of yearly rate estimates, data for patients <15 years of age are not included.

However, the overall rate was severalfold higher in persons >65 years of age than in persons ages 45–64 years, and the rate in persons ages 45–64 years was in turn higher than rates in persons ages 15–44 years and persons ages <15 years (Table). In addition, significant increasing trends were found between 2000 and 2003 in both of the older age groups; however, the slope of the increase was severalfold greater in those ages >65 years (*b* = 58.1, 95% CI 36.5–79.7, p = 0.01) than those ages 45–64 years (*b* = 7.9, 95% CI 4.0–11.7, p = 0.03) (Figure 2).

**Figure 3:**
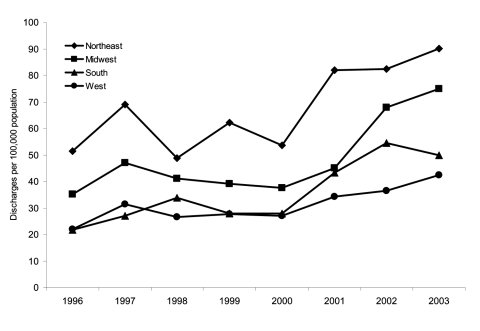
Rates of US short-stay hospital discharges with *Clostridium difficile* listed as any diagnosis, by region. Northeast (Connecticut, Maine, Massachusetts, New Hampshire, Rhode Island, Vermont, New Jersey, New York, Pennsylvania), Midwest (Indiana, Illinois, Michigan, Ohio, Wisconsin, Iowa, Kansas, Minnesota, Missouri, Nebraska, North Dakota, South Dakota), South (Delaware, Washington DC, Florida, Georgia, Maryland, North Carolina, South Carolina, Virginia, West Virginia, Alabama, Kentucky, Mississippi, Tennessee, Arkansas, Louisiana, Oklahoma, Texas), and West (Arizona, Colorado, Idaho, New Mexico, Montana, Utah, Nevada, Wyoming, Alaska, California, Oregon, Washington, Hawaii) regions as defined by US Census Bureau.

Regional rates of CDAD as any discharge diagnosis are shown in Figure 3. Although rates appeared to increase in each US region from 2000 to 2003, a significant linear trend was found only for the Midwest (*b* = 13.1, 95% CI 5.4–20.8, p = 0.04) and South (*b* = 7.9, 95% CI 3.4–12.3, p = 0.04). Overall, during 1996–2003, the rate of CDAD as any discharge diagnosis was higher in the Northeast than in the Midwest, South, and West (Table).

**Figure 4:**
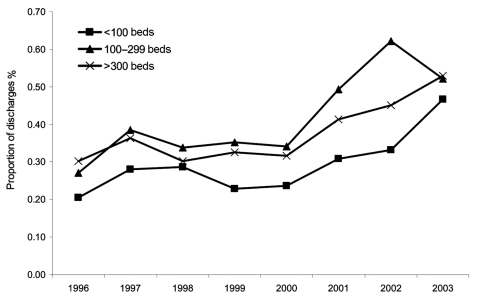
Proportion of US short-stay hospital discharges with *Clostridium difficile* listed as any diagnosis, by hospital size (number of beds).

The proportions of discharges, including CDAD as any diagnosis, stratified according to hospital size, are shown in Figure 4. Although rates appeared to increase in each group, from 2000 to 2003, a significant linear trend was found only for hospitals with 100 to 299 beds (*b* = 0.066, 95% CI 0.027–0.10, p = 0.04) and >300 beds (*b* = 0.070, 95% CI 0.04–0.10, p = 0.02). Overall rates for 1996–2003 were similar between hospitals with 100–299 beds and hospitals with >300 beds, whereas rates in hospitals with <100 beds were significantly lower than rates in either group of larger sized hospitals (Table).

The absolute number of CDAD patients who were transferred to a long-term care facility increased from 20,000 (95% CI 13,000–28,000) in 2000 to 57,000 (95% CI 43,000–71,000) (*b* = 13,000, 95% CI 8,000–17,000, p = 0.01). CDAD discharges also accounted for an increasing proportion of all patients who were transferred to long-term care facilities during this period, rising from 0.78% (95% CI 0.52–1.04) of long-term care transfers in 2000 to 1.87% (95% CI 1.48–2.26) in 2003 (*b* = 0.37, 95% CI 0.23–0.52, p = 0.01). Although the point estimate of CDAD inpatients who died before discharge increased from 8,000 (95% CI 5,000–12,000) in 2000 to 15,000 (95% CI 10,000–20,000 in 2003, this trend was not significant (*b* = 1,910, 95% CI 100–3,700, p = 0.1). Moreover, the proportion of deaths among CDAD discharges did not change significantly: 8.52% (95% CI 5.40–11.64) in 2000 to 8.39% (95% CI 6.09–10.69) in 2003 (*b* = 0.25, 95% CI 0.90–1.40, p = 0.70).

## Discussion

We found both the number and rate of US short-stay hospital discharges with a diagnosis of CDAD increased from 2000 through 2003. The overall rate and increase in the number of discharges were most prominent among patients >65 years of age. Although the overall rate was highest in the Northeast, a significant linear increase was found only in the Midwest and South. Rates increased similarly in middle- and large-sized hospitals according to number of beds, and overall rates were similar in these 2 strata; in contrast, small hospitals had a lower overall rate and did not experience a significant increase.

Our study had several limitations. We analyzed only hospital discharge data and the sensitivity and specificity of hospital discharge coding for CDAD are largely unknown (*16**,**17*). Although ICD-9-CM code for "an intestinal infection with *C*. *difficile*" contains the organism name, the code may be used on the basis of the clinical suspicion of CDAD alone, in some instances without a positive laboratory test result. In a single-institution study, when the number of positive *C*. *difficile* laboratory results were compared to the number of ICD-9-CM coded diagnoses, ICD-9-CM diagnoses overestimated by 32% the number of cases predicted by positive laboratory results (*16*). However, a month-to-month correlation was found between cases detected by a positive *C*. *difficile* laboratory test result and ICD-9-CM coded diagnosis; when patient medical records of these disparate cases (i.e., agreement with ICD-9-CM code and negative for *C*. *difficile* laboratory results or not tested) were reviewed in detail, most had a history of CDAD during a previous admission or had a *C*. *difficile* laboratory test ordered that either had a negative result or was cancelled before specimen collection. Another recent comparison of laboratory and ICD-9-CM data from all US Veterans Affairs hospitals demonstrated that ICD-9-CM coded diagnoses of CDAD underestimated by approximately half the number of CDAD patient discharges determined by positive laboratory results (*17*). These investigators also confirmed that ICD-9-CM coded diagnoses of CDAD correlated with the number of CDAD patient discharges determined by positive laboratory results, both among different hospitals as well as over time within individual hospitals.

Along with the potential insensitivity of coding, limitations involve the sensitivity of commonly used diagnostic tests for CDAD. Many hospital laboratories have migrated away from performing culture for *C*. *difficile* (sensitive but nonspecific for toxin-producing strains) or tissue culture cytotoxin assays toward the performance of less time-consuming, but generally less sensitive, toxin immunoassays. Data are available on diagnostic methods other than bacterial culture collected as part of proficiency surveys conducted by the College of American Pathologists (CAP) between 1999 and 2003 (*18*). Responses from at least 2,250 North American clinical laboratories surveyed annually suggest that, from 1999 through 2003, <5% laboratories overall performed tissue culture cytotoxin assays. Although this proportion is small, even if it were much larger, sensitivity could still be an issue as some evidence has shown that even a tissue culture cytotoxin assay performed directly on stool misses a large proportion of patients with diarrhea and low numbers of cytotoxin-producing *C*. *difficile (*19*)*.

One possible interpretation of our findings is that the observed increase in CDAD reflects a migration away from immunoassays that detect only toxin A toward use of assays that detect toxin A and B after reports of fatal cases associated with toxin A–negative and toxin B–positive strains (*20*). Indeed, the data from CAP mentioned above indicate such a trend: 78% of laboratories in 1999 performed toxin A immunoassays only and 7% performed toxin A and B immunoassays, whereas by 2003, 55% performed toxin A only immunoassays, and 38% performed toxin A and B immunoassays (*18*). Although this change in testing practices was gradual, even if it had been more sudden, the increased sensitivity of combined toxin A and B immunoassays could not likely explain the observed increase in rates between 2000 and 2003 because toxin A-B–positive strains account for no more than 5% of *C*. *difficile* isolates (*21*). Nonetheless, to address this issue as well as the possibility that more CDAD cases were being diagnosed due to increased *C*. *difficile* testing, we analyzed rates of diarrhea using ICD-9-CM codes for nonspecific causes and found no significant decrease. This suggests that the observed increases in CDAD were not simply due to diagnosing more disease among patients with diarrhea of nonspecific causes (data not shown).

Our findings suggest a large impact of excess illness and costs posed by CDAD on the US healthcare system; an impact that may approach or exceed that caused by other more widely recognized nosocomial pathogens. For example, the number of US short-stay hospital discharge diagnoses of CDAD during either 2001, 2002, or 2003 exceeded the estimated annual number (120,000) of methicillin-resistant *Staphylococcus aureus* (MRSA) infections for 1999–2000 (*22*). Kyne et al. recently estimated that each case of CDAD in their hospital was associated with $3,699 in excess healthcare costs and 3.6 extra days of hospitalization (*7*). Based upon their hospital's rate of CDAD in 0.7% of discharges, they estimated that the total excess in US healthcare costs attributable to CDAD was likely >$1.1 billion. Even using our lower estimate for the total US cases in 2003 (i.e., 0.51% or 178,000), CDAD can be estimated to have resulted in >$600 million in excess healthcare costs and >600,000 excess hospital days in nonfederal facilities. However, such estimates only account for resource use in short-stay hospitals. Even though some residents infected in long-term care facilities receive their treatment in short-stay facilities, these estimates of excess healthcare costs do not account for the infection control and medication costs incurred within long-term care facilities.

Although our findings are consistent with a recent analysis of CDAD rates in NNIS hospitals (*12*), our results highlight 2 new and unique developments in the epidemiology of this disease. Archibald et al. described a gradual increasing trend between 1987 and 2001 in ICU rates from hospitals with >500 beds and in hospitalwide rates from hospitals with <250 beds (*12*). We found a sharp increase in hospitalwide rates during 2001–2003 following steady rates from 1996 to 2000; this increase was similar in medium and large hospitals. In contrast to early results from NNIS during the spread of MRSA (*23*), when rates were highest in hospitals with the greatest number of beds, we found that overall rates of CDAD were similar in medium and large hospitals.

Several possible explanations may account for the increasing national rates of CDAD. One includes potentially new and evolving patterns of antimicrobial drug use, for example, use of the fluoroquinolones that have recently been implicated in outbreaks of CDAD (*9**,**24**,**25*). Another potential contributing factor is the promotion of alcohol-based, waterless, hand sanitizers as the primary means of hand hygiene over soap and water. Because alcohol is not sporicidal, alcohol-based, waterless hand sanitizers may not be as effective as soap and water in removing *C*. *difficile*; this factor has led to the recommendation that "during outbreaks of CDAD, washing hands with a nonantimicrobial [agent] or antimicrobial soap and water after removing gloves is prudent" (*26*).

One important possibility is the emergence of strains of *C*. *difficile* that are more fit and capable of causing transmission and disease. This emergence would not be unprecedented; in the early 1990s a strain of *C*. *difficile* that was clindamycin resistant, the so-called "J strain," caused outbreaks in at least 5 geographically diverse hospitals (*5**,**27*). Indeed, a recent report suggests that an emerging fluoroquinolone-resistant, epidemic strain of *C*. *difficile* has been responsible for hospital outbreaks in at least 6 US states (Georgia, Illinois, New Jersey, Maine, Oregon, Pennsylvania) since 2001 (*28*). This epidemic strain has continued to spread among additional US states (Connecticut, Florida, Massachusetts, Ohio, Texas), Canada, and Europe (*29*). Another recent report suggests this strain produces 16- and 23-fold more toxins A and B, respectively, than current nonepidemic strains (*30*). Depending on where the earliest outbreaks caused by this epidemic strain were reported, the higher overall rates in the northeastern United States may reflect the early spread of this strain (*9**,**10**,**28**,**29*). If this hypothesis proves correct, it suggests other regions of the United States are likely to observe continued increases as the strain continues to spread geographically.

One striking finding we report is the marked variation in CDAD rates among different age groups, with rates in persons >65 years of age several fold higher than rates in the next younger age group (45–64 years). Although the importance of advanced age as a risk for CDAD is not a new idea (*1**,**3*), this is the first report of national CDAD rates according to age group. Several possible reasons may explain this association between CDAD and age, not the least of which is increased exposure to healthcare facilities (including both acute and long-term facilities) and antimicrobial drugs. In addition, older persons may have decreased host defenses to protect them from CDAD. These conditions include decreased stomach acidity resulting from achlorhydria or a possibly increased use of medications such as histamine-2 receptor blockers or proton-pump inhibitors, medications that are becoming increasingly recognized in association with CDAD (*31**,**32*). Recent evidence also suggests the importance of a humoral immune response in protecting against CDAD after colonization (*33**–**37*). Thus, the decreased immune responsiveness commonly observed in older groups may be important in the development of CDAD in patients >65 years of age.

Contact precautions are recommended to prevent transmission of *C*. *difficile* in the healthcare setting (*38*). These consist of placing patients with CDAD in private rooms or cohorting CDAD patients together, using gloves and gowns for all patient contact, and either using disposable patient care equipment or cleaning such equipment between use with different patients. In addition, removing certain potential fomites, such as reusable electronic thermometers, from use in the general hospital patient population is important for controlling outbreaks (*1*). Limited data support enhanced environmental cleaning, especially of heavily contaminated patient care equipment. Clinicians should be aware of the importance of adhering to these precautions for containing transmission of CDAD in healthcare facilities. However, because antimicrobial drug use is the single most important patient risk factor, the clinician's primary responsibility in the control of CDAD lies in the area of judicious antimicrobial drug use (*1*).

In conclusion, the overall scope and magnitude of CDAD are great and may exceed those of other important hospital pathogens (e.g., MRSA), which suggests that *C*. *difficile* is one of the most common nosocomial pathogens. In addition, the financial costs and patient illness caused by CDAD in US short-stay hospitals appear substantial. Patients >65 years of age and those in intermediate- or larger-sized hospitals appear disproportionately affected. Because rates appear to have markedly increased during the first 3 years of this decade, new initiatives in the areas of surveillance, prevention, and control of CDAD are urgently needed. In the meantime, clinicians should be aware of the risk posed by CDAD in their hospitalized patients, remain cognizant of the importance of judicious antimicrobial drug use, and support infection control efforts for CDAD in the healthcare settings where they practice.
